# Talking to Teens about Pain: A Modified Delphi Study of Adolescent Pain Science Education

**DOI:** 10.1080/24740527.2019.1682934

**Published:** 2019-11-26

**Authors:** Hayley B. Leake, Lauren C. Heathcote, Laura E. Simons, Jennifer Stinson, Steven J. Kamper, Christopher M. Williams, Laura L. Burgoyne, Meredith Craigie, Marjolein Kammers, David Moen, Joshua W. Pate, Kimberley Szeto, G. Lorimer Moseley

**Affiliations:** a IIMPACT, University of South Australia, Adelaide, South Australia, Australia; b Department of Anesthesiology, Perioperative and Pain Medicine, Stanford University School of Medicine, Palo Alto, California, USA; c Child Health Evaluative Sciences, The Hospital for Sick Children, Toronto, Canada; d Lawrence S. Bloomberg Faculty of Nursing, University of Toronto, Toronto, Canada; e Institute for Musculoskeletal Health, University of Sydney, Sydney, New South Wales, Australia; f School of Medicine and Public Health, Hunter Medical Research Institute, University of Newcastle, New South Wales, Australia; g Children’s Anaesthesia, Women’s and Children’s Hospital, Adelaide, South Australia, Australia; h College of Medicine and Public Health, Flinders University, Adelaide, South Australia, Australia; i Melbourne School of Psychological Sciences, The University of Melbourne, Melbourne, Victoria, Australia; j Form Physiotherapy, Adelaide, South Australia, Australia; k Faculty of Medicine and Health Sciences, Macquarie University, Sydney, New South Wales, Australia

**Keywords:** Pain science education, pediatric pain, chronic pain, education

## Abstract

**Background**: Persistent pain is a prevalent condition that negatively influences physical, emotional, social and family functioning in adolescents. Pain science education is a promising therapy for adults, yet to be thoroughly investigated for persistent pain in adolescents. There is a need to develop suitable curricula for adolescent pain science education.

**Methods**: An interdisciplinary meeting of 12 clinicians and researchers was held during March 2018 in Adelaide, South Australia. An *a priori* objective of the meeting was to identify and gain consensus on key learning objectives for adolescent pain science education using a modified-Delphi process.

**Results and Conclusion**: Consensus was reached via a modified Delphi process for seven learning objectives to form the foundation of a curriculum: 1) Pain is a protector; 2) The pain system can become overprotective; 3) Pain is a brain output; 4) Pain is not an accurate marker of tissue state; 5) There are many potential contributors to anyone’s pain; 6) We are all bioplastic and; 7) Pain education is treatment. Recommendations are made for promising areas for future research in adolescent pain science education.

## RÉSUMÉ

**Contexte**: La douleur persistante est une pathologie répandue qui influence négativement le fonctionnement physique, émotionnel, social et familial chez les adolescents. L'éducation à la science de la douleur est une thérapie prometteuse pour les adultes, mais doit encore faire l'objet d’études plus approfondies en ce qui concerne la douleur persistante chez les adolescents. Il est nécessaire d'élaborer des programmes d'études appropriés pour l'éducation aux sciences de la douleur chez les adolescents.

**Méthodes**: Une réunion interdisciplinaire de 12 cliniciens et chercheurs s'est tenue en mars 2018 à Adélaïde, en Australie du Sud. L'un des objectifs de la réunion fixé *a priori* était de déterminer par consensus les principaux objectifs d'apprentissage de l'éducation à la science de la douleur chez les adolescents à l'aide d'un processus Delphi modifié.

**Résultats et conclusion**: Un processus Delphi modifié a permis d’atteindre un consensus sur les sept objectifs d'apprentissage qui devraient constituer la base d'un programme d'études : 1) La douleur est un protecteur ; 2) Le système de la douleur peut devenir surprotecteur ; 3) La douleur est un produit du cerveau; 4) La douleur n'est pas un marqueur précis de l'état des tissus ; 5) Il y a beaucoup de acteurs contributifs potentiels à la douleur de chaque personne; 6) Nous sommes biologiquement plastiques et; 7) L'éducation à la douleur est un traitement. Des recommandations sont formulées en ce qui concerne les domaines les plus prometteurs sur lesquels devraient porter les futures études en matière d’éducation des adolescents à la science de la douleur.

## Introduction

Pain is common in adolescents.^1,2^ For a significant minority, pain persists^3^ and negatively impacts physical, emotional, social and family functioning.^4–9^ Understanding why pain persists and how to respond effectively to it can be challenging for those who experience persistent pain. Guidelines recommend healthcare providers assist adolescents with persistent pain to understand their pain experience.^10^ An established form of education – *pain science education* – aims to provide a framework to understand one’s pain condition, by exploring what pain is, what function it serves, and how it works.^11^ Research suggests that when pain science education is delivered in conjunction with other treatments within a biopsychosocial framework, adults with persistent pain experience clinically meaningful improvements in pain and disability.^11–13^ However, little is known about the application or effectiveness of pain science education for adolescents with persistent pain.

Broadly, pain education can be divided into two components: pain *science* education (i.e. *how* and *why* is pain produced?), and pain *management* education (i.e. what should you *do* about your pain?). Pain *science* education aims to align a clinician and patient’s understanding of pain with modern pain science, whereby pain represents a need to protect, rather than a perceived reflection of tissue damage.^14,15^ Commonly pain science education involves changing one’s concept of pain from a biomedical paradigm toward a biopsychosocial model that considers the experience of pain to be influenced by biological (e.g. nociception, genetics), psychological (e.g. cognitions, beliefs), and social/contextual (e.g. family, school) factors.

Interdisciplinary care is recommended as the optimal model for treating adolescent persistent pain,^10^ however, many therapies offered in interdisciplinary care may appear counterintuitive to one’s understanding of the cause of their pain. For example, if someone with persistent pain believes their pain is an indicator of tissue damage, they may consider psychological therapies irrelevant, and limit movement or immobilize the painful body part to prevent further damage. One potential method to reverse this situation is to give people with pain a clear explanation of why psychological therapies and movement-based therapies are key strategies for persistent pain. As such, pain *science* education acts as a prelude to pain *management* education; it is not intended to replace active rehabilitation interventions, but rather to enhance and facilitate their acceptance. Additionally, improving one’s understanding of pain may influence the perception of pain itself, as demonstrated in studies with adults.^11,12^ This is predicted on the basis of contemporary theories of brain and neurological function (see Wallwork et al.^16^), which emphasize the capacity of cortical processing to integrate cognitive and contextual variables.^17,18^

There is limited evidence investigating pain science education in adolescents. To date, three school-based studies have demonstrated that adolescents have the capacity to learn pain science topics via a 30-minute lecture,^19^ an 11-minute video,^20^ and a four-week classroom-style intervention.^21^ The classroom intervention study is the only randomized controlled trial, investigating the effects of adolescent pain science education, by combining education with neck exercises for chronic neck pain.^21^ However, this study was too small to detect clinically-meaningful effects, and included a short follow-up (i.e. 4 weeks).

Curricula-building is an important part of health education, yet no curricula exist to educate adolescents about pain. We set out to begin the process of developing an adolescent pain science education curriculum, starting with establishing key learning objectives. An interdisciplinary meeting was held in March 2018 in Adelaide, South Australia. The *a priori* objective of the meeting was to identify and gain consensus on key learning objectives for adolescent pain science education using a modified-Delphi process.

## Methods

### Design

We conducted a three-round modified Delphi process during an interdisciplinary meeting on 20-21st March 2018 in Adelaide, Australia. The Delphi approach is a consensus method to determine the extent to which a group of individuals agree on given topics, using iterative rounds, interspersed with controlled feedback.^22^ A modification involved the fact that respondents were not anonymous during the discussion process.

### Panel

A convenience sample of participants were invited to attend the meeting if they were available to attend a workshop in Adelaide on 20-21st March 2018 following the Pain Adelaide Scientific Meeting on 19th March 2018, and satisfied one or more of the following criteria: previously published in the field of pain science education, pediatric pain or pain perception, working clinically in pediatric pain medicine, expertise in pain curriculum development or consumer-targeted pain education resources or pursing graduate studies in pediatric pain. There is no consensus regarding the optimal number of panelists in the Delphi-process; the experience and expertise of the panelists is considered more important than the number.^23^ G.L.M. invited panelists, coordinated the meeting and served as moderator.

### Procedures

On the first day, panelists attended a series of presentations to review the state of the field of pediatric pain science education (see Table 1 for meeting agenda). On day two, a modified Delphi study was conducted to identify and gain consensus on pain science education learning objectives for adolescents. The number of rounds was predetermined at 3. Panelists were instructed to focus on the content of the learning objective, not on developing age-appropriate language for that learning objective.

**Table 1.** Meeting agenda surrounding modified-Delphi.

Day 1
4 x presentations from panelists on topics including a child’s concept of pain, pediatric pain in public health, current pain education resources and designing pain management resources. Group discussion regarding: What is the current evidence base for adolescent pain education? Are there gaps in the evidence base? What are the barriers to adolescent pain education? Who are the target learners for pain education? Are there particular pain conditions for pain education? Consider a roadmap for funding, development, testing and dissemination of pain education resource. Identify key persons as peer review committee. Outline expectations and responsibilities moving forward. – Moderator collates findings from the discussion and provides to group via e-mail for review.
**Day 2** Recap of previous days findings. Commence modified Delphi-process to identify adolescent pain science learning objectives. Round 1 Round 2 Round 3 Discussion regarding next steps.

### Round 1

In round 1, panelists were divided into two groups by the moderator aiming to achieve an even split of expertise. Both groups were asked to list potential learning objectives for adolescent pain science education. As stimulus, both groups were provided a reference list of pain science learning objectives previously developed for adults^24,25^ and children (aged 8–12).^26^ The moderator compiled all suggested learning objectives into one list.

### Round 2

In round 2, the compiled list of proposed learning objectives was supplied to the two groups. The groups were asked to remove similar or duplicate learning objectives and those they deemed unnecessary. The moderator retrieved the two lists and noted discrepancies. A discussion around discrepancies was facilitated by the moderator, until consensus was achieved.

### Round 3

In round 3, the list of potential learning objectives derived from round 2 were presented to all panelists. They were asked to anonymously select the top five learning objectives for relevance to adolescents using anonymous, electronic survey software (SurveyMonkey™). After each round the anonymous results were reported to the panelists, and they were given the opportunity to “rescue” the bottom two ranked objectives. If a rescue was attempted, it would trigger a group discussion and a revote. If no rescue was attempted, the bottom two ranked objectives were discarded until seven remained. We chose seven objectives on the basis of a large literature, particularly the work of Miller.^27^ We concede that that work was based on short-term memory experiments, and that keeping to “the magical number seven” when it comes to learning objectives also reflects the collective opinion of panelists on a balance between coverage of the content and manageability of the curriculum.

## Results

### Panel

A total of 14 invitations were sent to potential participants. Twelve participants formed the panel, and all 12 participated in the entirety of the three-round modified-Delphi process. The characteristics of the participants are presented in Table 2. The mean age of the panelists was 38.3 years (± 12.4 years). Seven participants (58.3%) were female. Nine participants (75%) worked in Australia, two (16.7%) in the United States of America and one (8.3%) in Canada. The panel was constituted of experts from various health-related professions, including; physical therapists (50%), psychologists (25%), a medical doctor (8.3%), a nurse (8.3%) and an exercise physiologist (8.3%).

**Table 2.** Characteristics of the panel.

Panelists (n = 12)	N (%)
Age mean (SD)	38.3 (± 12.4)
Gender (female)	7 (58.3)
Education	
PhD	7 (58.3)
Master’s degree	3 (25)
Bachelor’s degree	2 (16.7)
Place of work*	
University or other research institute	9 (75)
Hospital	6 (50)
Primary care	3 (25)
Years of work experience, mean (SD)	
In research (n = 12)	7.9 (6.2)
In clinical practice (n = 9)	15.4 (13.2)
Professional background	
Medicine	1 (8.3)
Nursing	1 (8.3)
Psychology	3 (25)
physical therapy	6 (50)
Exercise physiology	1 (8.3)
Country of work	
Australia	9 (75)
United States of America	2 (16.7)
Canada	1 (8.3)
Expertise*	
Pediatric pain	8 (66.7)
Persistent pain	9 (75)
Pain science education	6 (50)
Pain perception	3 (25)
Pain curriculum development	5 (25)
Creating consumer-targeted pain education resources	3 (25)

*More than one option could be selected.

### Results of Round 1

**Figure 1.**
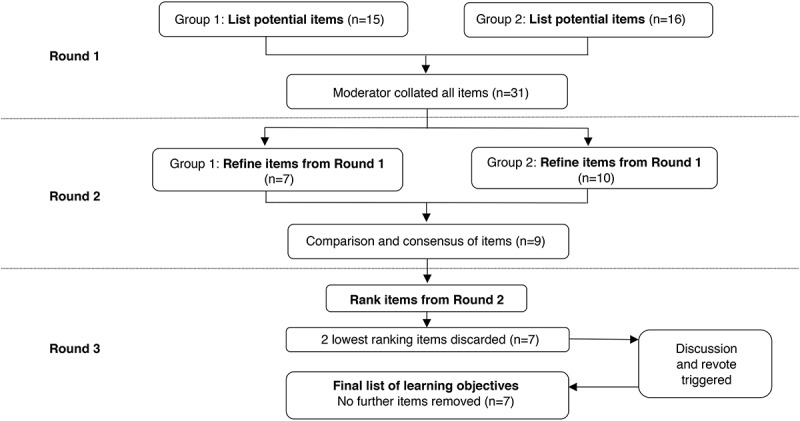
Flow chart of Delphi-style process for adolescent pain science education learning objectives. (n = number of learning objectives).

A flow chart outlining the modified-Delphi process is provided in Figure 1. In round 1, two groups of six panelists were formed. Group 1 and Group 2 proposed 15 and 16 learning objectives respectively, resulting in a total of 31 candidate learning objectives for adolescent pain science education.

### Results of Round 2

The same two groups of six panelists were retained for round 2. Following group discussions, Group 1 and Group 2 retained 7 and 10 learning objectives respectively. The moderator then facilitated a discussion with all panelists to reach consensus on discrepancies. The discrepancies included: (1) Group 2 split *“pain is a protective output”* into two learning objectives: *“pain is a protector”* and *“the pain system can become overprotective”*. Consensus was reached that these would remain 2 separate learning objectives. (2) Group 2 included *“it is possible to live a normal life again”* as an independent learning objective, whereas Group 1 removed this objective entirely. Agreement was reached to include this objective, but re-worded to *“it is possible to improve/get better/you can do it”*. (3) Group 2 had included *“no-one else can decide whether or not you are in pain”* as an independent learning objective, whereas Group 1 clustered this in a group titled *“your pain is unique and real”*. Agreement was reached to amalgamate the two objectives into one, reworded to *“your pain is unique and real (valid) and no-one else can decide if you have it”*. (4) The following objectives did not fall under another category and both groups agreed on removing these entirely: *“your brain is not broken”* and *“pain is common”*. At the conclusion of round 2, consensus was reached that 9 of the 31 original learning objectives were retained.

### Results of Round 3

**Figure 2.**
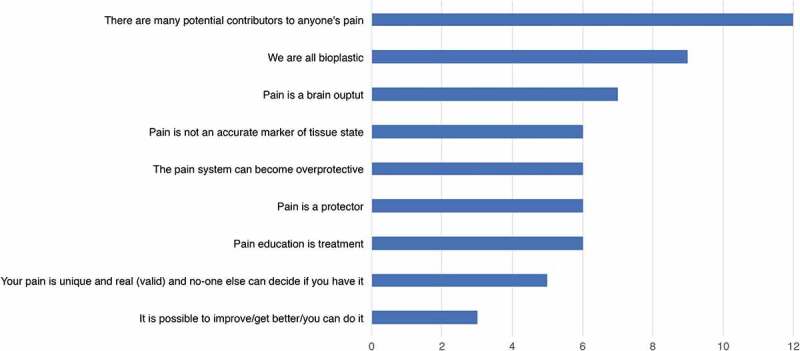
Outcome of round 3 ranking of top five adolescent pain science learning objectives by 12 panelists.

Panelists indicated the top five most relevant learning objectives for adolescents, from the nine identified in round 2 (see Fig. 2). The highest ranked learning objective was *“there are many potential contributors to anyone’s pain”* indicated in the top 5 by all 12 participants (100%). The two lowest ranked objectives were *“pain is unique and real (valid) and no-one can decide if you have it”* (5 of 12; 42%) and *“it is possible to improve/get better/you can do it”* (3 of 12; 25%). A re-vote and discussion were triggered, resulting in the same two learning objectives being ranked lowest, and consequently discarded. At the conclusion of round 3 the following seven learning objectives were proposed: 1) Pain is a protector; 2) The pain system can become overprotective; 3) Pain is a brain output; 4) Pain is not an accurate marker of tissue state; 5) There are many potential contributors to anyone’s pain; 6) We are all bioplastic and; 7) Pain education is treatment. Further explanations of adolescent learning objectives are presented in Table 3. A comparison of adult and adolescent pain science learning objectives is presented in Table 4.

**Table 3.** Key learning objectives for adolescent pain science education resulting from a modified-Delphi style consensus.

Learning objective	Meaning
*Pain is a protector*	The purpose of pain is protection, not detection of damage. The protective purpose of pain integrates evidence showing a range of factors from across biopsychosocial domains that modulate pain. The protective purpose of pain integrates the effect of inflammation on stimulus response profiles of primary nociceptive afferents and the effect of enhanced response profiles within nociceptive processing in the spinal cord and brain (see Moseley & Butler 2018^25^ for expanded review).
*The pain system can become overprotective*	A reduction in response thresholds (allodynia), increase in receptive fields^28^ and a widening of effective stimuli reflect an enhancement of the protective function of pain. This concept includes the notion that the longer pain persists, the more likely it is that it is overprotective.
*Pain is a brain output*	Pain is not created in the tissues but is a conscious feeling that urges one to act to protect a particular body part or parts. While an isolated brain could not produce pain, the brain is the most proximal and major contributor to the experience.
*Pain is not an accurate marker of tissue state*	Experimental and clinical data clearly demonstrate that pain does not hold an isomorphic relationship with tissue state, nor nociceptive activity.^29,30^
*There are many potential contributors to anyone’s pain*	Pain is a biopsychosocial phenomenon. Contributions to pain are personally unique, influenced by previous exposure and learning, and context dependent. Other factors that influence pain include emotional state, sleep, nutrition, physical state, understanding of pain, other sensory cues (see Moseley & Butler 2018^25^ for expanded review).
*We are all bioplastic*	Biological systems are inherently adaptive and change in function and often in structure in response to demand. Learning within the pain system can explain enhanced sensitivity, reduced pain thresholds and hyperalgesia that accompany many persistent pain states.^18^ Active and targeted strategies can reduce sensitivity of the pain system (e.g.^31,32^)
*Pain education is treatment*	Evidence based guidelines for treating pain internationally recommend education as firstline intervention. There is Level 1 evidence from adult studies that demonstrate clinical benefits of pain education.^11,12^ The notion that pain-related knowledge influences pain is consistent with contemporary theories in the pain field.

**Table 4.** A comparison of adolescent and adult learning objectives for pain science education.

Adolescent	Adult	
**Modified-Delphi results**	**Explain Pain Supercharged**^25^	**Revised Neurophysiology of Pain Questionnaire**^24^
Pain is not an accurate marker of tissue damage	Pain and tissue damage rarely relate	Pain only occurs when you are injured or at risk of being injured (False)
		Chronic pain means that an injury hasn’t healed properly (False)
		Worse injuries always result in worse pain (False)
		Pain occurs whenever you are injured (False)
Pain is a brain output	Pain involves distributed brain activity	The brain decides when you will experience pain (True)
There are many potential contributors to anyone’s pain	Pain relies on context	When you injure yourself, the environment that you are in will not affect the amount of pain you experience, as long as the injury is exactly the same (False)
Pain education is treatment	Learning about pain can help the individual and society	
We are all bioplastic	We are bioplastic	
Pain is a protector	Pain is one of many protective outputs	
Pain can become overprotective		
	Pain is normal, personal and always real	
	Active treatment strategies promote recovery	
	Pain depends on the balance of danger and safety	
	There are danger sensors, not pain sensors	When part of your body is injured, special pain receptors convey the pain message to your brain (False)
		Special nerves in your spinal cord convey “danger” messages to your brain (True)
		When you are injured, special receptors convey the danger message to your spinal cord (True)
		It is possible to have pain and not know about it (False)
		Nerve adapt by increasing their resting level of excitement (True)
		Descending neurons are always inhibitory (False)

## Discussion

Following a modified-Delphi process 12 panelists reached consensus on seven key learning objectives for adolescent pain science education: 1) Pain is a protector; 2) The pain system can become overprotective; 3) Pain is a brain output; 4) Pain is not an accurate marker of tissue state; 5) There are many potential contributors to anyone’s pain; 6) We are all bioplastic and; 7) Pain education is treatment. These learning objectives have the potential to form the basis of an education curriculum to improve an adolescent’s knowledge of pain.

It is noteworthy to consider that a large proportion of panelists (43%) ranked the learning objective *“pain is unique and real (valid) and no-one else can decide if you have it”* in their list of top five objectives. However, due to our *a-priori* aim of retaining only seven learning objectives, this item was not included in the final outcome. Some may consider this eighth learning objective clinically useful and we include it here for consideration of future endeavors in adolescent pain science education.

### Comparison between Adult and Adolescent Learning Objectives

There are similarities between extant adult and new adolescent pain science learning objectives (see Table 4). Both sets of objectives emphasize the involvement of the brain in pain perception, differentiate pain from tissue damage or injury, and outline that the pain system can adapt. However, there are also clear differences. The adult version outlines neurophysiological processes of pain persistence (e.g. descending modulation), for adolescents, the concept that *“pain can become overprotective”* is introduced instead. The adolescent concepts are also simplified compared to adult educational objectives. For example, while adult objectives refer to distributed brain activity, the adolescent objectives describe pain as a brain output, and state that *“pain is a protector”*. Instead of describing the influence of environmental or contextual factors as for adults, the adolescent objectives state *“there are many potential contributors to anyone’s pain”*. Finally, the adolescent objectives do not include language that refers to “pain messages” versus “danger messages”, such is seen in adult objectives.

It is important to keep in mind that the aim of the Delphi process was to identify the broad concepts, not precisely define the optimal wording. The language used to present these concepts to adolescents will likely need to be simplified and embedded in examples and contextual information. The differences between adult and adolescent learning objectives for pain science likely reflects the panelists views on what constitutes developmentally appropriate content for adolescence. Achieving language appropriate for adolescent development was outside the scope of the aim of this meeting, however addressing this will be a necessary next step.

### Limitations

This study has limitations. First, the panel did not include adolescents with persistent pain or their families, because we were concerned about developing the curriculum and key scientific concepts. We consider it imperative that adolescents and their families be involved in progressing from this stage to the development of resources, clarification of messages, and identification of concepts they value and believe are important. As a next step, young people and their parents should be involved in the co-creation and testing of pain science education resources. Second, the panel included institutional representation from three countries, Australia, the United States and Canada. As such, the panelist’s reflections on learning objectives are likely to be influenced by the culture, ethnicity, and health care systems of these countries (although we note that some of the panelists had previously lived and grown up in other countries). It is possible that adolescents living in developing countries may require a different set of learning objectives. Third, this study did not differentiate developmental stages across adolescence. There may be benefit in tailoring pain science learning objectives to the different developmental stages (e.g. early, middle, late adolescence), rather than the entire period. Tailoring may be required according to an adolescent’s educational and literacy level. Future testing of these objectives could explore whether this is necessary.

### Future Directions

Several pain science learning objectives have been recommended, but a curriculum is required to extend this work. Future research may consider developing the content of these learning objectives, exploring the necessity of education tailored to pain conditions, testing delivery methods, and evaluating credibility of information. It is possible that adolescents with persistent pain may benefit from increasing pain science literacy within their wider community, including parents, caregivers, siblings, healthcare providers, school personnel and peers. There is an outstanding need for a validated tool to measure pain science knowledge of adolescents, such as those that exist for adults,^15,24^ and are being developed for children (8– 12 years).^26^ Finally, to determine what effect pain science education actually has on patient-relevant outcomes, trials should follow. Considering the difficulties undertaking randomized controlled trials in pediatric populations,^33^ alternate designs may be considered such as single case experimental designs and multiple-baseline designs.

## Conclusion

The interdisciplinary meeting on adolescent pain science education, held in Adelaide, Australia, gathered clinical and research professionals across pain education and pediatric pain to identify learning objectives for clinical application of pain science education to adolescents. We reached consensus on seven learning objectives to form the foundations of a pain science education curriculum: 1) Pain is a protector; 2) The pain system can become overprotective; 3) Pain is a brain output; 4) Pain is not an accurate marker of tissue state; 5) There are many potential contributors to anyone’s pain; 6) We are all bioplastic and; 7) Pain education is treatment.

## Supplementary Material

Supplemental Material
